# Influence Mechanism of Drug–Polymer Compatibility on Humidity Stability of Crystalline Solid Dispersion

**DOI:** 10.3390/ph16121640

**Published:** 2023-11-22

**Authors:** Chunhui Hu, Qiuli Yan, Yong Zhang, Haiying Yan

**Affiliations:** 1 State Key Laboratory of Plateau Ecology and Agriculture, Qinghai University, Xining 810001, China; 2 Medical College, Qinghai University, Xining 810001, China; zhongxueping2021@163.com (Q.Y.); zhangyong20100507@163.com (Y.Z.); tangshizhen199805@163.com (H.Y.)

**Keywords:** crystalline solid dispersion, humidity stability, compatibility, microstructure, dissolution behavior

## Abstract

This study investigates the influence of humidity on the dissolution behavior and microstructure of drugs in crystalline solid dispersions (CSDs). Using Bifonazole (BFZ) as a model drug, CSDs were prepared through spray drying with carriers such as Poloxamer 188 (P188), Poloxamer 407 (P407), and polyethylene glycol 8000 (PEG8000). The solubilization effect and mechanism were initially evaluated, followed by an examination of the impact of humidity (RH10%) on the dissolution behavior of CSDs. Furthermore, the influence of humidity on the microstructure of CSDs was investigated, and factors affecting the humidity stability of CSDs were summarized. Significant enhancements in the intrinsic dissolution rate (IDR) of BFZ in CSDs were observed due to changes in crystalline size and crystallinity, with the CSD-P188 system exhibiting the best performance. Following humidity treatment, the CSD-P407 system demonstrated the least change in the IDR of BFZ, indicating superior stability. The CSD-P407 system was followed by the CSD-P188 system, with the CSD-PEG8000 system exhibiting the least stability. Further analysis of the microstructure revealed that while humidity had negligible effects on the crystalline size and crystallinity of BFZ in CSDs, it had a significant impact on the distribution of BFZ on the CSD surface. This can be attributed to the water’s potent plasticizing effect, which significantly alters the molecular mobility of BFZ. Additionally, the compatibility of the three polymers with BFZ differs, with CSD-P407 > CSD-P188 > CSD-PEG8000. Under the continuous influence of water, stronger compatibility leads to lower molecular mobility and more uniform drug distribution on the CSD surface. Enhancing the compatibility of drugs with polymers can effectively reduce the mobility of BFZ in CSDs, thereby mitigating changes caused by water and ultimately stabilizing the surface composition and dissolution behavior of drugs in CSDs.

## 1. Introduction

The advancement of combinatorial chemistry, high-throughput screening technology, and rational design has led to the identification of an increasing number of active pharmaceutical ingredients (APIs). However, effective evaluation of drug developability has been lacking, resulting in a gradual rise in molecular weight [1,2,3]. For small-molecule drugs, poor drug developability often stems from low solubility [4]. Currently, approximately 40% of marketed drugs and nearly 90% of compounds under development worldwide belong to BCS Class II or IV, characterized by their low solubility [5]. Consequently, enhancing the solubility and oral bioavailability of insoluble drugs has become a crucial research area in the pharmaceutical field.

Among the various techniques available for improving drug solubility, solid dispersions (SDs) have garnered significant attention. SDs can be classified into amorphous solid dispersions (ASDs) and crystalline solid dispersions (CSDs) based on whether the drugs crystallize within a polymeric matrix [6,7]. In ASDs, the drug is dispersed in an amorphous state within the polymer matrix. Although amorphous drugs exhibit increased solubility and dissolution rates due to their high Gibbs free energy, they tend to undergo thermodynamic crystallization during storage, resulting in the loss of their solubilization advantages [8,9,10]. In contrast, CSDs offer several advantages over ASDs: (1) the drug exists in a crystalline form, providing improved thermodynamic stability compared to ASDs, thereby facilitating controllable production and storage; (2) for drugs prone to strong crystallization tendencies, such as albendazole [11] and *β*-Lapachone [12], CSDs offer unique solubilization advantages when other techniques, such as ASDs or cyclodextrin saturation technology, prove suboptimal.

The crystallization mechanism, stability and microstructure of CSD were systematically studied in the early stage, and it was found that steric hindrance, effective glass transition temperature and intermolecular interaction could affect the crystallization rate and crystalline size of drugs in CSD, thus affecting the dissolution rate of drugs. Hu et al. [11] prepared albendazole-poloxam 188 CSD by the spray drying method. The results showed that the growth of ABZ in CSD was affected by the steric hindrance of P188 crystal, the crystalline size of the drug was significantly reduced, and the dissolution rate of the drug was significantly increased. Zhang et al. [13] found that the effective glass transition temperature in a ketoconazol-poloxam 188-CSD system could regulate the nucleation and growth rate of drugs through temperature, thereby reducing the drug particle size to improve the dissolution rate of drugs. Huang et al. [14] found that in a CSD system of different flavonoids and poloxam 188, the stronger the molecular interaction between drug and carrier, the greater the reduction in drug particle size, which can effectively improve the dissolution rate of drugs. 

At present, most studies on the stability of solid dispersion (CD) are focused on amorphous drugs, and the influencing factors mainly include: (1) Different preparation methods: there are significant differences in the stability of amorphous drugs prepared by different methods. The factors that lead to these differences include the residual composition of the crystal nucleus in the amorphous form (it is easy to induce crystallization in the crystal nucleus), the free energy state of an amorphous state, and the arrangement and orientation of microscopic molecules in amorphous drugs [15,16,17]. (2) Properties of drugs: Trasi et al. found that amorphous compounds that are more prone to rapid crystallization usually have smaller relative molecular mass, higher melting point and density, and lower melting entropy and viscosity [18]. (3) External environment: storage environment factors such as temperature and humidity conditions will affect the stability of amorphous drugs. Empirical rules show that when the storage temperature of ASD preparation is much lower than its glass transition temperature (a difference of more than 50 ℃), the molecular mobility of amorphous drugs is very slow and usually has excellent physical stability [19]. Humidity conditions, such as water, act as a potential plasticizer; if liquid is absorbed by undissolved amorphous drugs, glass transition temperature is reduced, molecular mobility is enhanced, and it is easy for recrystallization to occur, resulting in the loss of the original solution-increasing effect [20]. However, the current research on CSD mainly focuses on its physical and chemical properties, microstructure, and dissolution behavior, and few scholars have conducted systematic research on whether the above stability factors are applicable to CSD.

To address this knowledge gap, we selected bifonazole (BFZ), a water-insoluble imidazole antifungal drug, as a model drug for this study. CSDs were prepared by spray drying, utilizing poloxamer 188 (P188), poloxamer 407 (P407), and polyethylene glycol 8000 (PEG8000) as carriers. Firstly, we examined the solubilization effect and mechanism of the three polymers on BFZ. Secondly, we evaluated the changes in CSDs at 25 °C/RH10%, considering both apparent phenomena such as dissolution rate and microstructure parameters like crystalline size, crystallinity, and surface composition. Finally, we elucidated the influence of humidity on the dissolution behavior and microstructure of CSDs, while discussing the molecular mechanisms affecting the microstructure and dissolution behavior of CSDs in relation to the compatibility of drugs within the polymeric matrix.

## 2. Results and Discussion

### 2.1. Methodological Validation

The selectivity of the established HPLC method for analyzing BFZ was confirmed using the HPLC chromatograms. The precision, repeatability, and stability of the method had residual standard deviations (RSD) of 1.96%, 1.99%, and 0.34%, respectively. The recovery of BFZ at low, middle, and high concentrations was 100.24%, 101.18%, and 99.86%, respectively, with corresponding RSD values of 0.06%, 0.18%, and 0.79%. The linear regression equation for BFZ was Y = 32.833X + 4.5123, with an R^2^ value of 0.9996, and the linear concentration range was 0.396–198.000 μg/mL. 

### 2.2. Characterization of CSD Prepared by P188, P407 and PEG8000

#### 2.2.1. Surface Morphology and Microstructure of CSD

**Figure 1:**
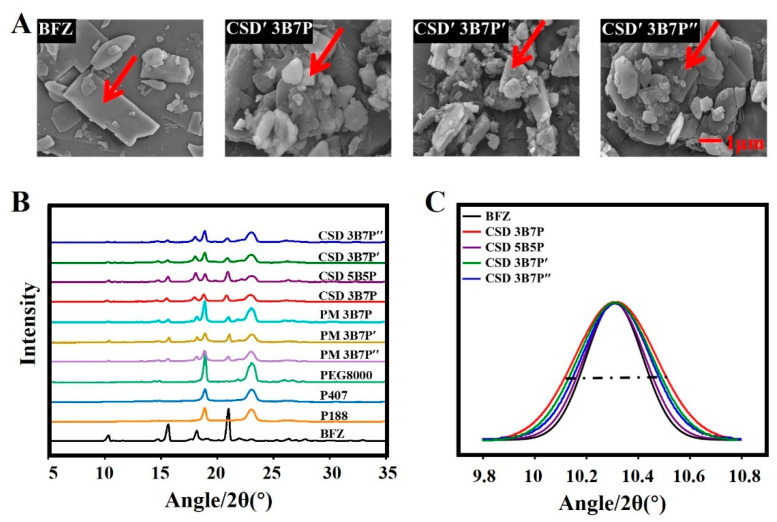
Characterization of CSD formulation. (**A**) Macroscopic SEM images, where 3B7P was 30% of the drug loading of CSD and red arrows represent BFZ; (**B**) PXRD profiles of BFZ and CSD; (**C**) Peak broadening effect in PXRD profiles. (scale bar for all pictures is 1.0 μm). B: BFZ, P: P188, P’: P407, and P″: PEG8000; 3B7P (P′, P″) and 5B5P respectively represent a 30% and 50% drug loading of CSD.

SEM is a commonly used method and characterization tool for observing the surface morphology, particle size, and distribution of drugs. For insoluble drugs, reducing the drug particle size can effectively improve the dissolution rate. Therefore, it is proposed to use scanning electron microscopy to observe the particle size and surface morphology of drugs in CSD (Figure 1A). Raw BFZ exhibited irregular flaky crystallization with a smooth surface and a crystalline size of about 5 μm. After washing the polymers from the CSD with deionized water, the surface morphology and crystalline size of BFZ in CSD were visually observed. The crystalline size of BFZ in the three types of CSD was smaller compared to raw BFZ, with an approximate size of 1 μm. The surfaces became rough, and CSD with P188 as the carrier showed the most significant reduction in crystalline size. According to the Ostwald ripening effect [21], the BFZ crystalline size observed in CSD would be larger during the washing process, but it remained smaller than that of raw BFZ. This indicates that the crystalline size of BFZ in CSD prepared with P188, P407, and PEG8000 was significantly reduced. According to the Noyes–Whitney equation (Equation (1)), a decrease in drug particle size increases the effective surface area of the drug particles, thereby significantly increasing the dissolution rate of the drug.

(1) $$\frac{dW}{dt}=\frac{D\cdot A\cdot \left(C_{s}-C\right)}{L}$$

*dW*/*dt* is dissolution rate; *D* is drug diffusivity; *A* is surface area; *C_s_* is saturated solubility of the drug; *C* is drug solubility in bulk solution; *L* is thickness of diffusion layer.

#### 2.2.2. Crystalline Form and Crystalline Domain Size (CDSz)

The PXRD diffraction patterns of BFZ and CSD with different polymeric matrices and drug loadings exhibited characteristic crystalline peaks at 2θ values of 10.31°, 15.62°, 18.18°, and 20.98° (Figure 1B). Aside from the characteristic peaks of the polymers, the characteristic peaks of CSD coincided with those of BFZ, indicating that the drug in the prepared CSD did not undergo crystal transformation. The average CDSz of BFZ in CSD was determined using the Scherrer equation (Figure 1C). The PXRD peak of the crystalline drug displayed significant broadening. The CDSz of raw BFZ was 34.64 ± 1.67 nm. When the drug loading was 30%, the average CDSz of BFZ in CSD with P188, P407, and PEG8000 as carriers was 31.93 ± 2.67 nm, 32.63 ± 3.13 nm, and 32.74 ± 3.54 nm, respectively, indicating a significant reduction in CDSz of BFZ.

#### 2.2.3. Crystalline Size of BFZ

**Figure 2:**
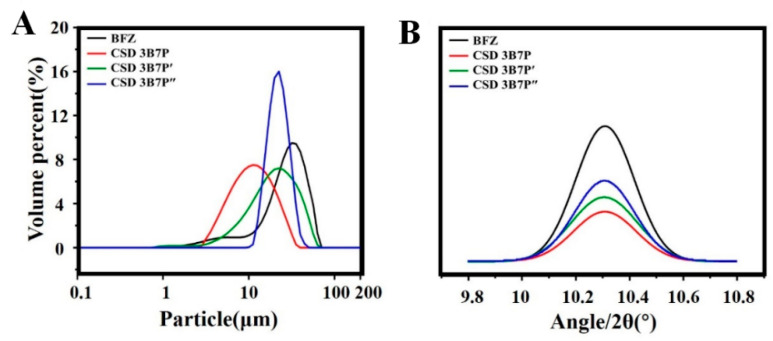
Particle size distribution profiles (**A**) and diffraction peak intensities (**B**) of BFZ-CSD and raw BFZ. B: BFZ, P: P188, P′: P407, and P″: PEG8000; 3B7P (P′, P″) respectively, represent 30% drug loading of CSD.

The average crystalline size of BFZ-CSD was smaller than that of the raw drug (Figure 2A, Table 1). The crystalline size of BFZ was 26.84 μm, and the average crystalline size of BFZ in different BFZ-CSD formulations was smaller than that of the raw BFZ. Among the formulations, CSD preprepared with P188 as the carrier exhibited the smallest crystalline size compared to that prepared with PEG8000 or P407. It should be noted that the measured complex of BFZ and polymer in this study included the polymer, so the crystalline size of the CSD was smaller than that of the raw BFZ, indicating a reduction in the crystalline size of BFZ in the CSD. The polydispersity coefficients were 0.205 (BFZ), 0.477 (CSD 3B7P), 0.206 (CSD 3B7P′), and 0.125 (CSD 3B7P″), respectively. Figure 2A represents the volume size distribution (%) of dispersed phase particles. A significantly larger fraction is occupied by the sample CSD 3B7P″, indicating that the drug particle size in CSD prepared using PEG8000 as the carrier is more uniform.

#### 2.2.4. Crystallinity of BFZ

DSC was used to measure the ∆H_f_ of raw BFZ and BFZ-CSD (Table 2). The ∆*H_f_* of raw BFZ was 109.95 J·g^−1^. In the CSDs, as the drug loading decreased, the ∆H_f_ of BFZ continued to decrease. The relative crystallinity (R_C_) of BFZ in CSD supported by P188, P407, and PEG8000 decreased from 100% to 9.91%, 11.91%, and 15.01%, respectively. 

It should be noted that while DSC can provide information on the crystallinity of drugs, PXRD is a non-destructive technique that allows qualitative and quantitative analysis of drugs without causing damage to crystal samples. Despite requiring a larger sample volume, PXRD provides relatively accurate results. For example, Figure 2B shows the diffraction peaks of drugs in CSD determined by PXRD. The diffraction peak intensities of CSDs were significantly lower compared to raw BFZ, with the weakest intensity observed in CSD-BFZ-P188. Previous studies have reported that the interaction between drugs and polymeric matrices can affect the phase behavior and crystallization process of drug–polymer hybrid systems.

A stronger interaction between the drug and polymeric matrices can delay the drug’s crystallization and reduce its crystallinity, which will be discussed further in the compatibility study.

#### 2.2.5. Intrinsic Dissolution Rate

Intrinsic dissolution rate (IDR) refers to the mass of a certain amount of drug dissolved in a specific medium per unit area of drug per unit time. It is an important parameter for the physicochemical properties of drugs and represents an inherent characteristic of drugs. It explains the large data dispersion observed in dissolution experiments caused by factors such as solvation, crystal form changes, and variations in crystallinity due to the drug itself.

**Figure 3:**
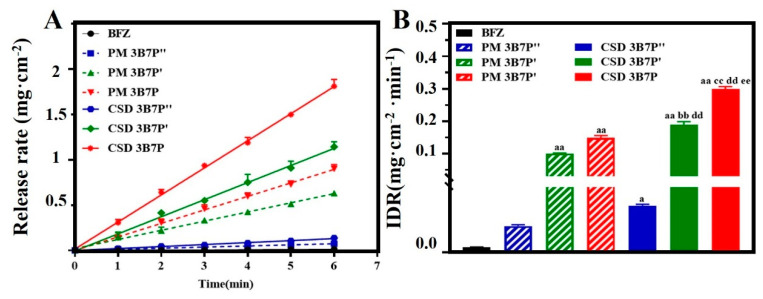
Intrinsic dissolution profiles (**A**) and intrinsic dissolution rate plots (**B**) of BFZ-CSD and raw BFZ ($\bar{x}\;\pm \;s$, n = 3). PM: physical mixing; B: BFZ, P: P188, P′: P407, and P″: PEG8000; 3B7P (P′, P″) respectively represent 30% drug loading of CSD. Compared with pure BFZ, a: *p* < 0.05; aa: *p* < 0.01; Compared with PM 3B7P′, bb: *p* < 0.01; Compared with PM 3B7P, cc: *p* < 0.01; Compared with CSD 3B7P″, dd: *p* < 0.01; Compared with CSD 3B7P′, ee: *p* < 0.01.

Figure 3A,B demonstrates that the IDR of drugs in BFZ-PM and BFZ-CSD increased compared to raw BFZ. CSD had a more pronounced effect on the IDR of drugs, with CSD-BFZ-P188 (0.2958 mg·cm^−2^·min^−1^) exhibiting the highest IDR, followed by CSD-BFZ-P407 (0.1896 mg·cm^−2^·min^−1^), PM-BFZ-P188 (0.1488 mg·cm^−2^·min^−1^), PM-BFZ-P407 (0.0971 mg·cm^−2^·min^−1^), CSD-BFZ-PEG8000 (0.0221 mg·cm^−2^·min^−1^), PM-BFZ-PEG8000 (0.0122 mg·cm^−2^·min^−1^), and BFZ (0.0024 mg·cm^−2^·min^−1^). The dissolution behavior of BFZ in BFZ-CSD was further improved compared to BFZ-PM, indicating that the wetting effect of polymers can also influence drug dissolution behavior. It was found that the wettability of polymers, crystalline size, and crystallinity of drugs collectively affect the dissolution behavior of BFZ.

### 2.3. Effect of Humidity on the Microstructure and Dissolution Behavior of CSD

#### 2.3.1. Effect of Humidity on the IDR

**Figure 4:**
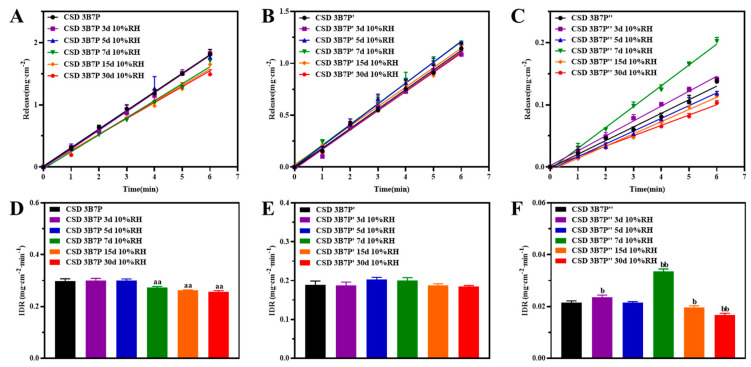
Intrinsic dissolution curves (**A**–**C**) and intrinsic dissolution rates (**D**–**F**) of CSD placed under humidity conditions ($\bar{x}\pm \;s,\;n=3$). B: BFZ, P: P188, P′: P407, and P″: PEG8000; 3B7P (P′, P″) respectively represent a drug loading of 30% for CSD, 3. 5, 7, 15, and 30 d represents the number of days in the humidity condition. Compare with CSD 3B7P, aa: *p* < 0.01; Compare with CSD 3B7P″, b: *p* < 0.05; bb: *p* < 0.01.

The IDR of CSD-BFZ with different carriers treated at RH10% for various durations exhibited differences (Figure 4A–C). The IDRs of CSD-BFZ-P188 at 0, 3, 5, 7, 15, and 30 days were 0.2988, 0.3011, 0.2991, 0.2733, 0.2618, and 0.2562 mg·cm^−2^·min^−1^, respectively. The IDRs at 7, 15, and 30 days were statistically significant compared to day 0 (*p* < 0.05) (Figure 4D). The IDRs of CSD-BFZ-P407 at the same time points were 0.1885, 0.1878, 0.2022, 0.1997, 0.1874, and 0.1848 mg·cm^−2^·min^−1^, respectively (Figure 4E). The IDRs of CSD-BFZ-PEG8000 were 0.0216, 0.0236, 0.0214, 0.0335, 0.0196, and 0.0167 mg·cm^−2^·min^−1^, respectively. The IDRs at 3 and 15 days were statistically significant compared to day 0 (*p* < 0.05), while the IDRs at 7 and 30 days were significantly different from day 0 (*p* < 0.01) (Figure 4F).

The results indicated that the dissolution behavior of CSD with P188 and PEG8000 as carriers became unstable after treatment at 25 °C/RH10%. Dissolution behavior, as a macroscopic phenomenon, indirectly reflects changes in the microstructure of CSD, such as crystalline size, crystallinity, and surface concentration. In this study, we systematically investigated the relevant factors in the microstructure of the BFZ and polymers to determine which factors undergo changes induced by humidity, thereby affecting the dissolution behavior of the drug.

#### 2.3.2. Effect of Humidity Treatment on the Crystallinity and Microstructure of CDSz

**Figure 5:**
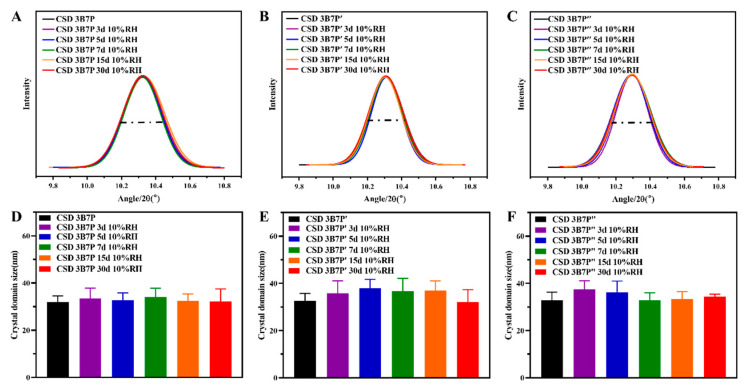
CSD peak broadening effect (**A**–**C**) and BFZ domain size (**D**–**F**) under humidity conditions ($\bar{x}\;\pm \;s,\;n=3$). B: BFZ, P: P188, P′: P407, and P″: PEG8000; 3B7P (P′, P″), respectively, represent a drug loading of 30% for CSD; 3, 5, 7, 15, and 30 d represents the number of days in the humidity condition.

After subjecting BFZ-CSD to RH10% treatment for different durations, peak broadening of the drug’s characteristic peaks was determined using PXRD (Figure 5A–C). The peak broadening of BFZ in CSD prepared with the three polymers did not change with treatment time, and the CDSz of BFZ in CSD did not show significant changes (*p* < 0.05) (Figure 5D–F).

**Figure 6:**
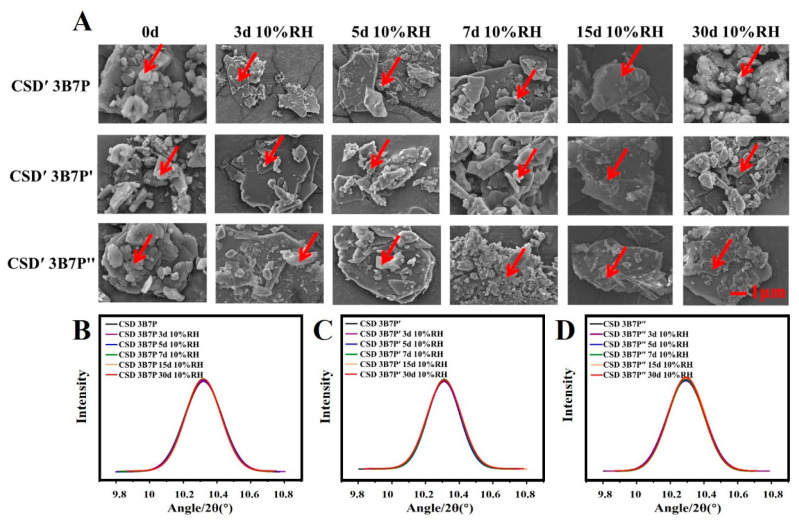
The micro-morphology (**A**) and crystallinity of BFZ in CSD under humidity conditions (**B**–**D**) (B: BFZ, P: P188, P′: P407, and P″: PEG8000; 3B7P (P′, P″), respectively, represent a drug loading of 30% for CSD; 3, 5, 7, 15, and 30 d represents the number of days in the humidity condition. Red arrow refers to the crystalline BFZ.

SEM was utilized to observe the micro-morphology of BFZ-CSD after RH10% treatment. As depicted in Figure 6A, the surface roughness of BFZ in CSD′-BFZ-P188 and CSD’-BFZ-P407 did not change significantly. Over time, the surface of BFZ in CSD’-BFZ-PEG8000 became rough, and the crystalline size showed a slight change.

The crystallinity of BFZ in CSD following RH10% treatment was determined using PXRD (Figure 6B–D). The peak intensity of BFZ in CSD-BFZ-P188 and CSD-BFZ-P407 did not change, indicating that the crystallinity remained unaffected. Compared to day 0, the peak intensity of BFZ in CSD-BFZ-PEG8000 at different time points showed a slight increase, indicating a slight elevation in crystallinity, although not statistically significant (*p* < 0.05). Therefore, humidity did not significantly affect the crystallinity of BFZ in CSD.

#### 2.3.3. Compatibility of BFZ with Polymers

In evaluating the compatibility of drugs with polymers, parameters such as the Hansen solubility parameter, Flory–Huggins interaction parameter, and drug solubility in polymers are commonly used. 

**Figure 7:**
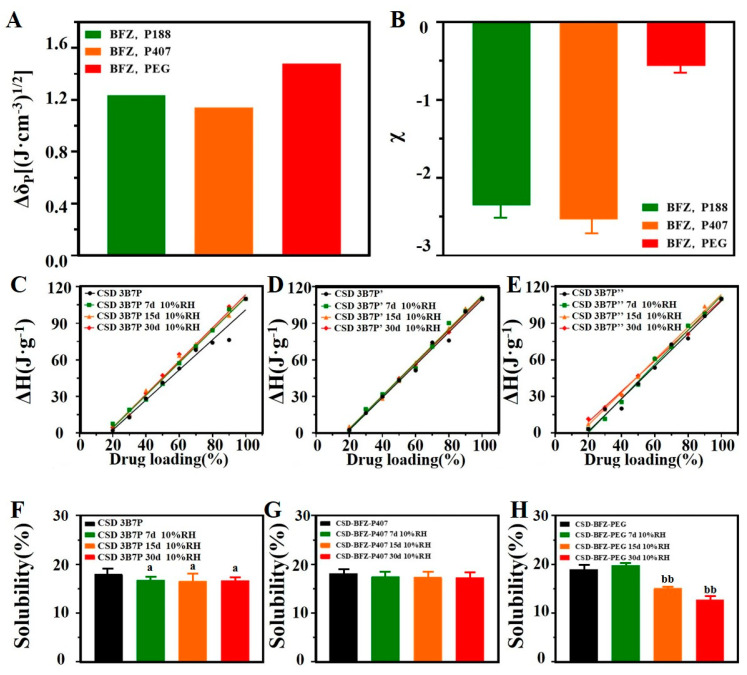
Compatibility of BFZ with polymers. (**A**) Hansen solubility parameter of BFZ with different polymers; (**B**) Flory–Huggins interaction parameter of BFZ with different polymers ($\bar{x}\;\pm \;s,\;n=3$) [23]; (**C**) Solubility curve of BFZ in P188; (**D**) Solubility curve of BFZ in P407; (**E**) Solubility curve of BFZ in PEG8000; (**F**–**H**) Solubility of BFZ in P188, P407 and PEG8000 ($\bar{x}\;\pm \;s,\;n=3)$. B: BFZ, P: P188, P’: P407, and P″: PEG8000; 3B7P (P’, P’’), respectively, represent a drug loading of 30% for CSD. Compare with CSD 3B7P, a: *p* < 0.05; Compare with CSD 3B7P″, bb: *p* < 0.01.

The Hansen solubility parameter, developed by Hansen in 1967, predicts the solubility and solution formation between molecules. It quantitatively expresses the principle of “similar solubility [22].” In our study, the solubility parameters of BFZ were compared with those of P188, P407, and PEG8000, yielding ∆δp (BFZ, P188) = 1.2335 (J·cm^−3^)^1/2^, ∆δp (BFZ, P407) = 1.1418 (J·cm^−3^)^1/2^, and ∆δp (BFZ, PEG8000) = 1.4805 (J·cm^−3^)^1/2^, respectively (Figure 7A). BFZ exhibited the best compatibility with P407, followed by P188, while PEG8000 showed the least compatibility.

The Flory–Huggins interaction parameter (χ-Value) is used to determine the strength of interaction between drugs and polymers. For drug–polymer systems, a negative χ-value (χ < 0) indicates a stronger interaction between drugs and polymers compared to drug–drug or polymer–polymer interactions, while a positive χ-value (χ > 0) suggests weak or no interaction [23]. In our study, the χ-values for BFZ with P188, P407, and PEG8000 were −2.3555, −2.5381, and −0.5685, respectively (Figure 7B). All χ-values were negative, indicating the existence of interaction between BFZ and the three polymers. The interaction between BFZ and P407 was the strongest, followed by P188, while PEG8000 exhibited the weakest interaction.

DSC is a method used to quantitatively measure the solubility of pharmaceutical compounds dispersed in polymeric matrices. The principle of this method is based on the fact that the fraction of solubilized drug within the matrix should not contribute to the melting endotherm associated with the dispersed drug fraction. By calculating the Δ*H_f_* in a series of drug–polymer dispersions and plotting Δ*H_f_* versus drug loading, the solubility of the drug within the matrix can be evaluated [24,25]. Figure 7C–E presents the plot of Δ*H_f_* versus drug loading for solid dispersion (SD) samples with drug loadings ranging from 20% to 90% (*w*/*w*). 

As shown in Table 3, the solubility of BFZ in P188 on P188 on days 0, 7, 15, and 30 of humidity treatment was 18.03%, 16.83%, 16.64%, and 16.66%, respectively. There was a statistically significant difference at 7, 15, and 30 days compared to day 0 (*p* < 0.05). The solubility of BFZ in P407 was 18.17%, 17.55%, 17.39%, and 17.29%, respectively. The solubility of BFZ in PEG8000 was 19.02%, 19.77%, 15.12%, and 12.72%, respectively. There was a significant difference at 15 and 30 days compared to day 0 (*p* < 0.01). These findings are consistent with the conclusions regarding the strength of drug–polymer compatibility.

#### 2.3.4. Surface Composition of CSD

**Figure 8:**
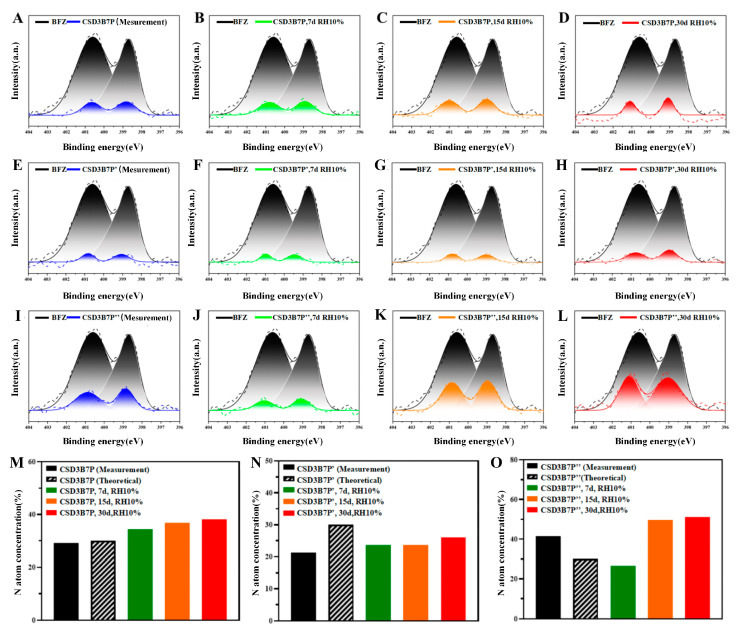
XPS spectra of BFZ and percentage of surface concentration of BFZ in CSD placed under humidity conditions. (**A**–**D**) XPS spectra of CSD-BFZ-P188 on days 0, 7, 15, 30; (**E**–**H**) XPS spectra of CSD-BFZ-P407 on days 0, 7, 15, 30; (**I**–**L**) XPS spectra of CSD-BFZ-PEG8000 on days 0, 7, 15, 30. Comparison of XPS Spectral Specific Data of CSD, P188 (**M**), P407 (**N**), and PEG8000 (**O**) as Carriers. B: BFZ, P: P188, P′: P407, and P″: PEG8000; 3B7P (P′, P″), respectively, represent a drug loading of 30% for CSD.

The drug–polymer compatibility ultimately affects the surface composition of drugs on CSD, thereby influencing their dissolution behavior. Figure 8 presents several representative XPS spectra for pure BFZ and BFZ/polymer CSD. Each spectrum exhibits two peaks corresponding to the photoelectrons from the 1s orbital of a nitrogen atom linked to a benzene ring (401 eV) or carbon (399 eV). These two nitrogen atoms are labeled N (C_6_H_5_-C-N) and N (C=N-C). In the BFZ and BFZ/polymer spectra (black peaks), the two peaks have nearly equal intensities, indicating a 1:1 stoichiometric ratio between the two nitrogen atoms in a molecule. The colored peaks represent the BFZ-CSD spectrum after different durations of RH10% treatment. The ratio of the BFZ-CSD peak to the BFZ peak is used to calculate the surface concentration of BFZ.

In the BFZ/P188 system, the surface drug concentration was consistent with the theoretical value (30% drug loading) (Figure 8M). After 7, 15, and 30 days of humidity treatment, the surface concentration of BFZ exceeded 30%, increasing by 5.25%, 7.56%, and 8.84%, respectively. This led to surface enrichment and drug precipitation, resulting in a decrease in the IDR of CSD-BFZ-P188. In the BFZ/P407 system, the drug concentration on the surface was lower than the theoretical value, indicating a smaller drug fraction at the particle surface compared to the bulk (Figure 8N). After humidity treatment, the surface concentration of BFZ slightly increased, but did not exceed 30%. The surface composition of BFZ remained uniform, and the dissolution behavior of CSD-BFZ-P407 remained unchanged. In the BFZ/PEG8000 system, the drug fraction at the particle surface was larger than in the bulk (Figure 8O). After humidity treatment, the surface concentration of BFZ exhibited a trend of initial decrease followed by an increase, with changes of −15.11%, 8.07%, and 9.61%. This resulted in an unstable drug distribution of BFZ on the surface, leading to changes in the dissolution behavior of CSD-BFZ-PEG.

We first observed significant deviations between the surface concentration of drugs and the bulk concentration. This deviation could be higher (CSD-BFZ-PEG8000) or lower (CSD-BFZ-P407) depending on the specific drug–polymer combination. This may be attributed to surface tension, where components with lower surface tension tend to accumulate on the surface, and vice versa. Secondly, the intervention of water could cause changes in the drug concentration on the surface of CSD. This may be due to the formation of a drug–polymer–water three-phase system during humidity treatment. In this ternary system, water acts as a powerful plasticizer, increasing the molecular mobility of BFZ in CSD. However, the compatibility between drugs and polymers limits the molecular mobility of drugs. Lastly, under the continuous influence of water, stronger compatibility between drugs and polymers results in lower molecular fluidity and a more uniform distribution of drugs on the CSD surface. Since the model drug BFZ used in this study is water-insoluble, a higher concentration of BFZ on the surface of CSD leads to a lower IDR. This explanation effectively clarifies why CSD prepared with different polymers exhibit distinct dissolution behaviors under the same humidity treatment.

## 3. Materials and Methods

### 3.1. Materials

**Figure 9:**
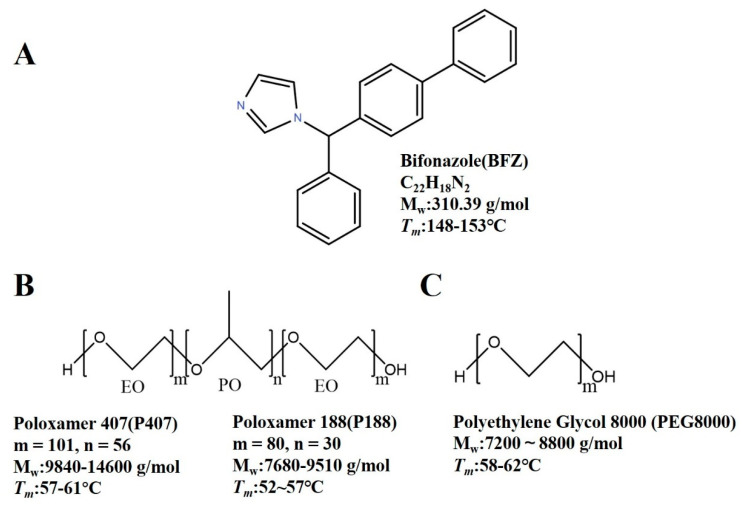
Structure of (**A**) bifonazole (BEZ), (**B**) poloxamer 407 (P407) and poloxamer 188 (P188), and (**C**) polyethylene glycol 8000 (PEG8000).

Bifonazole (BFZ, CAS: 60628-96-8), poloxamer 407 (P407, CAS: 9003-11-6), poloxamer 188 (P188, CAS: 9003-11-6), and polyethylene glycol 8000 (PEG8000, CAS: 25322-68-3) (purity > 98%) were obtained from Beijing Coupling Technology Co., Ltd. (Beijing, China). Methanol of high-performance liquid chromatography (HPLC) grade was purchased from Merck Company (Darmstadt, Germany). Potassium phosphate and other reagents were of analytical grade and obtained from Tianjin Damao Chemical Reagent Technology Co., Ltd. (Tianjin, China). The chemical structures and related physicochemical properties of the drugs and polymers used in the experiment are shown in Figure 9.

### 3.2. HPLC Method and Methodology

The drug concentration of BFZ was determined using HPLC (Agilent 1260 Series, Palo Alto, Santa Clara, CA, USA) with UV detection at 242 nm. A Diamonsil C18 column (4.6 × 250 mm) was used, and the mobile phase consisted of methanol/water (80/20 *v*/*v*) with a flow rate of 1 mL/min. The HPLC method was validated for specificity, calibration curve, precision, repeatability, stability, and recovery [13].

### 3.3. Preparation of BFZ-CSD and Physical Mixing (PM)

Samples of drugs and polymers with different mass ratios were accurately weighed with a total mass of 1.00 g and completely dissolved with 20 mL CH_2_Cl_2_ at a total concentration of 5% (*w*/*v*), the prescriptions for CSD in Table 4. The CSDs were prepared using a spray dryer (Yamato spray dryer ADL 311S, Yamato Scientific Company, Ltd., Santa Clara, CA, USA) The spray dryer operated with an inlet temperature of 52 °C, an outlet temperature of 32 °C, a solution feed rate of 8 mL/min, and an atomizing N_2_ pressure of 0.1 MPa. After spray drying, the SDs were vacuum dried for at least 24 h and stored in a desiccator at room temperature. Pure BFZ and pure polymers were prepared following the same procedure. The PM of BFZ and polymers was achieved by sieving and thoroughly mixing the two sample powders to ensure uniform distribution. 

### 3.4. Powder X-ray Diffraction (PXRD) 

PXRD patterns were obtained using an ESCALAB™ XI+ X’pert Powder X-ray Diffractometer (ESCALAB™ XI+, Waltham, MA, USA). The samples were placed in a zero-background silicon sample holder. PXRD experiments were conducted with an automatic divergence slit graphite monochromator (0.2 mm receiving slit). Scans were performed continuously from 5° to 35° (2θ) at a rate of 1°/min using a scanning step size of 0.01° 2θ. The diffraction peak was calculated using the Scherrer equation (Equation (2)), which determines the crystalline domain size (CDSz) of the crystallite perpendicular to the (102) reflection. The Scherrer equation used is as follows:

(2) τ=K λ/(βτ cosθ) 

where τ is the average crystallite dimension perpendicular to the (hkl) reflection, K is a shape factor (typical value of about 0.9), λ is the X-ray wavelength (1.54 Å), β_τ_ is the line broadening at half the maximum intensity, and θ is the Bragg angle.

### 3.5. Scanning Electron Microscopy (SEM) 

The surface morphology of the CSD was assessed using SEM (JSM-7900F, Tokyo, Japan) at an excitation voltage of 10 kV. Samples were placed on a copper platform and coated with gold for 180 s prior to observation.

### 3.6. Differential Scanning Calorimetry (DSC) 

#### 3.6.1. Drug–Polymer Flory–Huggins Interaction Parameter

The solubility of the crystalline drug in the polymeric matrix was determined using an annealing method developed by Tao [26]. Drug–polymer mixtures were annealed at various temperatures to achieve phase equilibrium and then scanned via DSC to detect residual drug crystals. The upper and lower bounds of the equilibrium solution temperature for each drug–polymer mixture annealed at different temperatures were obtained. The drug activity at a given solubility can be calculated using Equation (3). The drug–polymer interaction parameters were calculated using the Flory–Huggins model shown in Equation (4).

(3) $${\ln\alpha }_{d}=\Delta H_{m}/R\left({1/T}_{m}-1/T\right)$$

(4) $${\ln\alpha }_{d}{=\ln\Phi }_{d}+\left(1-1/x\right)\Phi_{p}{+\chi \Phi }_{p}^{2}$$

where α_d_ is the drug activity, Tm is the melting temperature of the pure drug, ΔH_m_ is the molar heat of fusion of the pure drug, T is the solubility temperature, Φ_d_ is the volume fraction of the drug, Φ_p_ is the volume fraction of the polymer, x is the molar volume ratio of the polymer and the drug, and χ is the drug–polymer interaction parameter. 

#### 3.6.2. Compatibility of BFZ with Polymers

DSC was used to determine the melting point (T_m_) and melting enthalpy (∆H_f_) using a STA449F3-DSC200F3 (Netzsch, Germany). Samples weighing 5–10 mg were loaded into pin-holed crimped aluminum pans and heated from 20 °C to 240 °C at a rate of 10 °C/min. The ∆H_f_ value was calculated by integrating the area under the melting endotherm. The solubility of drugs in different polymeric matrices was evaluated by extrapolating the melting enthalpy of drugs with varying drug loads to the intersection point with the abscissa, which represents the solubility of drugs in the polymeric matrix [24,25].

### 3.7. Hansen Solubility Parameter

The Hansen solubility parameter method, which predicts drug–polymer miscibility based on calculated solubility parameters (Δδp), was used to determine compatibility between molecules [27]. The interaction parameters were calculated using the cohesive energy density (Equation (5)). The total solubility parameters of drugs and polymers were calculated using Equation (6). The partial solubility parameters of a substance were calculated using the Group Contribution Method (Equation (7))

(5) $${\delta =(\mathrm{CED})}^{0.5}=(\Delta E_{V}{/V}_{m}{)}^{0.5}$$

(6) $$\delta_{t}{=\delta }_{d}^{2}{+\delta }_{p}^{2}{+\delta }_{h}^{2}$$

(7) $$\delta_{d}=\frac{\sum^{\;}F_{d}}{V};\delta_{p}=\frac{\sqrt{\sum^{\;}F_{p}^{2}}}{V};\delta_{h}=\sqrt{\frac{\sum^{\;}E_{h}}{V}}$$

where ΔE_v_ is the evaporation energy, V_m_ is the molar volume of the substance, δ_p_, δ_h_, and δ_d_ represent the solubility parameters of dispersion, polarity, and hydrogen bonding, F_d_ is the molar absorption constant of the dispersion group, F_p_ is the molar absorption constant of the polar group, E_h_ is the hydrogen bonding energy, and V is the molar volume of the substance.

### 3.8. Laser Particle Size Analyzer

The crystalline size of CSD was determined using a Mastersizer 2000 (Malvern Instruments, Malvern, UK) in dry test mode. Approximately 1.0 g of the sample was added to the dry test sample cell. A refractive index (RI) of 1.00 was used for the dispersion medium. The RI of 1.616 was used for BFZ, and the absorption index of 0.01 was used. The sample detection duration was 10 s, and the dispersion pressure was 3.0 bar. The injection speed was 50%, the slit width was 1.5 mm, and the shading was set between 1.0% and 5.0%.

### 3.9. Stability Experiment

Precision-weighed amounts of 2.0 g BFZ-CSD sample were placed in sealed containers at 10% RH. After 3, 5, 7, 15, and 30 days, the samples were removed and freeze-dried for 48 h to remove residual water before conducting PXRD, IDR, and other characterization to evaluate the differences in physicochemical properties.

### 3.10. X-ray Photoelectron Spectroscopy (XPS)

XPS was employed to detect the surface elemental compositions of solid dispersion particles using an ESCALab 250Xi (Thermo Scientific, Waltham, MA, USA) equipped with 200 W monochromatic Al Kα radiation. The base pressure in the analysis chamber was approximately 3 × 10^−10^ mbar. SAXPS analysis used a 500 μm X-ray spot, and the hydrocarbon C1s line at 284.8 eV from adventitious carbon was used for energy referencing. Spectral analysis was conducted using Thermo Avantage v5.9921 software (Thermo Scientific, Waltham, MA, USA), and curve fitting was performed using Origin 2022 software. The relative atomic concentrations in blends were calculated based on the integral peak intensities and sensitivity factors provided by the manufacturer. The fraction of each component at the surface was calculated from the chemical formulas and the characteristic elements of the drug and polymer.

### 3.11. Intrinsic Dissolution Rate (IDR)

To compare the IDR of BFZ and CSDs, an intrinsic dissolution method was used. Raw BFZ and CSDs before and after stability testing were compacted into sharp cylindrical tablets (11 mm diameter and ~2 mm thickness). The tablets weighed 80 mg and had a tablet hardness of 5 Kgf, measured using a tablet hardness tester. The tablets were placed into syringe tubes using paraffin, and a single surface was exposed to 20 mL of a dissolution medium in a 25 mL beaker. The dissolution medium consisted of 0.01 M HCl (pH = 1.4), and the temperature was maintained at 37 °C with stirring at 100 rpm (n = 3). At 0.5-min intervals, 0.3 mL of the solution was removed, centrifuged at 15,000 rpm for 3 min, double diluted with methanol, and assayed for drug concentration using HPLC. The IDR was calculated using Equation (8). The IDR equation used is as follows:

(8) $$\mathrm{IDR}=C\;V/(\pi \;r^{2}\;t)\;$$

where IDR is intrinsic dissolution rate, C is concentration of drug in solution; V is solution volume, r is the radius of the tablet, and t represents time. 

### 3.12. Statistical Method

SPSS 22.0 was used for statistical analysis. The measurement data with normal distribution were expressed by $\bar{x}\;\pm \;s$. One-way ANOVA was used for inter-group comparisons, and the LSD *t*-test was used for multiple comparisons. *p* < 0.05 means the difference is statistically significant.

## 4. Conclusions

In this study, CSD-BFZ prepared with P188, P407, and PEG8000 as carriers demonstrated no crystal transformation, with reductions observed in the domain size, crystalline size, and crystallinity of BFZ within the CSD. Notably, the use of P188 as a carrier had the most pronounced impact on these properties, resulting in improved drug dissolution behavior in CSD-BFZ-P188. Additionally, treatment of BFZ-CSD at RH 10% allowed for analysis of the intrinsic dissolution rate of CSDs. The results indicated that CSD-BFZ-P407 exhibited the most stable dissolution, followed by CSD-BFZ-P188, while CSD-BFZ-PEG8000 showed the largest difference in dissolution behavior. The microstructure of CSD revealed negligible changes in crystalline size and crystallinity of drugs across different CSDs; however, significant alterations were observed in the drug composition on the surface. Further investigations into drug–polymer compatibility unveiled that the system compatibility ranged as follows: P407-BFZ > P188-BFZ > PEG8000-BFZ. The greater the compatibility, the more uniform the drug distribution on the CSD surface and the more stable the dissolution behavior. Compared to ASD, drugs in CSD exist in crystalline form, and theoretically, their thermodynamic stability is much greater than ASD, making them relatively controllable in production, storage, and use. However, no scholars have systematically studied which factors affect the stability of CSD and how they affect it. This study confirms that water, acting as a potential plasticizer, substantively increases the molecular mobility of drugs, counteracting the forces generated by drug polymer compatibility. Under the continuous influence of water, stronger compatibility results in lower molecular mobility and more uniform plug distribution on the CSD surface. The possible factors affecting the stability of CSD were confirmed through intermolecular interactions and the influence of water on stability. It is once again proven that water may be one of the main factors affecting the stability of ASD or CSD, providing a theoretical basis for the design, preparation, and storage of CSD in the future.

## Figures and Tables

**Table 1:** Crystalline size of BFZ-CSD and BFZ.

Group (30% Drug Loading)	Crystalline Size (μm)		
	Dv (0.1)	Dv (0.5)	Dv (0.9)
BFZ	8.10	26.84	45.44
CSD-BFZ-P188	5.45	11.56	23.29
CSD-BFZ-P407	7.10	18.76	38.04
CSD-BFZ-PEG8000	14.55	20.64	29.33

**Table 2:** The ∆*H_f_* and relative crystallinity BFZ-CSD and raw BFZ.

Drug Loading (%)	P188		P407		PEG8000	
	∆H_f_ (J·g^−1^)	R_C_ (%)	∆H_f_ (J·g^−1^)	R_C_ (%)	∆H_f_ (J·g^−1^)	R_C_ (%)
100 (raw BFZ)	109.95	100.00	109.95	100.00	109.95	100.00
90	84.77	77.10	110.63	100.62	106.51	96.87
80	92.56	84.19	94.83	86.24	96.95	88.18
70	97.29	88.48	105.99	96.39	103.81	94.42
60	88.17	80.19	85.58	77.84	89.25	81.17
50	82.42	74.96	86.54	78.71	80.84	73.52
40	70.60	64.21	74.75	67.99	50.33	45.77
30	42.87	38.99	54.60	49.66	65.60	59.66
20	10.90	9.91	13.10	11.91	16.50	15.01

**Table 3:** Solubility of BFZ in CSD placed under RH10%.

CSD	Time	Linear Equation of Δ*H*_f_-Drug Loading	R^2^	Solubility
CSD-BFZ-P188	0 d	Y = 1.2331X − 22.234	0.9718	18.03 ± 0.62%
	7 d	Y = 1.3345X − 22.453	0.9946	16.83 ± 0.41%
	15 d	Y = 1.3367X − 22.248	0.9935	16.64 ± 0.82%
	30 d	Y = 1.3614X − 22.681	0.9920	16.66 ± 0.22%
CSD-BFZ-P407	0 d	Y = 1.3362X − 24.274	0.9903	18.17 ± 0.12%
	7 d	Y = 1.3629X − 23.923	0.9940	17.55 ± 0.15%
	15 d	Y = 1.3639X − 23.722	0.9970	17.39 ± 0.18%
	30 d	Y = 1.3424X − 23.205	0.9985	17.29 ± 0.23%
CSD-BFZ-PEG	0 d	Y = 1.3371X − 25.437	0.9877	19.02 ± 0.89%
	7 d	Y = 1.4005X − 27.683	0.9939	19.77 ± 0.22%
	15 d	Y = 1.3377X − 20.231	0.9968	15.12 ± 0.11%
	30 d	Y = 1.2517X − 15.924	0.9962	12.72 ± 0.35%

**Table 4:** Prescriptions for CSD with different drug loadings.

Drug Loading	CH_2_Cl_2_/mL	BFZ/g	Polymer/g
30%	20	0.3	0.7
50%	20	0.5	0.5

## Data Availability

The datasets used or analyzed during the current study are available from the corresponding author upon reasonable request.
